# Epidemiological study on *Listeria monocytogenes* in Egyptian dairy cattle farms’ insights into genetic diversity of multi-antibiotic-resistant strains by ERIC-PCR

**DOI:** 10.1007/s11356-022-19495-2

**Published:** 2022-03-17

**Authors:** Mona M. Elsayed, Rasha M. Elkenany, Amira I. Zakaria, Basma M. Badawy

**Affiliations:** 1 Department of Hygiene and Zoonoses, Faculty of Veterinary Medicine, Mansoura University, Mansoura, 35516 Egypt; 2 Department of Bacteriology, Mycology and Immunology, Faculty of Veterinary Medicine, Mansoura University, Mansoura, 35516 Egypt; 3 Department of Food Hygiene and Control, Faculty of Veterinary Medicine, Mansoura University, Mansoura, 35516 Egypt

**Keywords:** Antibiotic resistance, Dairy cattle farm, ERIC-PCR, *Listeria monocytogenes*, Molecular epidemiology, Risk factors

## Abstract

*Listeria monocytogenes* (*L. monocytogenes*) is frequently detected in ruminants, especially dairy cattle, and associated with the sporadic and epidemic outbreak of listeriosis in farms. In this epidemiological study, the prevalence, virulence, antibiotic resistance profiles, and genetic diversity of *L. monocytogenes* in three Egyptian dairy cattle farms were investigated. The risk factors associated with the fecal shedding of *L. monocytogenes* were analyzed. The *L. monocytogenes* strains from the three farms were categorized into distinct genotypes based on sampling site and sample type through enterobacterial repetitive intergenic consensus polymerase chain reaction (ERIC-PCR). A total of 1896 samples were collected from animals, environments, and milking equipment in the three farms. Results revealed that 137 (7.23%) of these samples were *L. monocytogenes* positive. The prevalence of *L. monocytogenes* in the animal samples was high (32.1%), and the main environmental source of prevalent genotypes in the three farms was silage. For all sample types, *L. monocytogenes* was more prevalent in farm I than in farms II and III. Risk factor analysis showed seasonal variation in production hygiene. For all sample types, *L. monocytogenes* was significantly more prevalent in winter than in spring and summer. The level of *L. monocytogenes* fecal shedding was high likely because of increasing age, number of parities, and milk yield in dairy cattle. Two virulence genes, namely, *hlyA* & *prfA*, were also detected in 93 strains, whereas only one of these genes was found in 44 residual strains. Conversely, *iap* was completely absent in all strains. The strains exhibited phenotypic resistance to most of the tested antibiotics, but none of them was resistant to netilmicin or vancomycin. According to sample type, the strains from the animal samples were extremely resistant to amoxicillin (95.2%, 80/84) and cloxacillin (92.9%, 78/84). By comparison, the strains from the environmental samples were highly resistant to cefotaxime (86.95%, 20/23). Furthermore, 25 multi-antibiotic resistance (MAR) patterns were observed in *L. monocytogenes* strains*.* All strains had a MAR index of 0.22–0.78 and harbored antibiotic resistance genes, including extended-spectrum β-lactamase (*bla*CTX-M [92.7%] and *bla*DHA-1 [66.4%]), quinolones (*qnrS* [91.2%], *qnrA* [58.4%], *parC* [58.4%], and *qnrB* [51%]), macrolides (*erm*[B] [76.6%], *erm*(C) [1.5%], and *msr*(A) [27%]), trimethoprim (*dfrD* [65.7%]), and tetracyclines (*tet*(M) [41.6%], *tet*(S) [8%], and *int-Tn* [26.3%]). ERIC-PCR confirmed that the strains were genetically diverse and heterogeneous. A total of 137 isolated *L. monocytogenes* strains were classified into 22 distinct ERIC-PCR groups (A–V). Among them, ERIC E (10.2%) was the most prevalent group. These results indicated that environment and milking equipment served as reservoirs and potential transmission ways of virulent and multidrug-resistant *L. monocytogenes* to dairy animals, consequently posing threats to public health. Silage is the main environmental source of prevalent genotypes on all three farms. Therefore, hygienic measures at the farm level should be developed and implemented to reduce *L. monocytogenes* transmission inside dairy cattle farms.

**Supplementary Information:**

The online version contains supplementary material available at 10.1007/s11356-022-19495-2.

## Introduction

*Listeria monocytogenes* is a facultative intracellular Gram-positive bacterium, which has been widely explored because of its association with numerous outbreaks of listeriosis worldwide (Vazquez-Boland et al. 2001). It is included in the World Health Organization list of foodborne pathogens and mostly related to raw milk, unpasteurized milk, and other dairy products (Shamloo et al. 2019). *L. monocytogenes* infections in humans and animals include eye infections, uveitis, keratitis (Nightingale et al. 2004), septicemia, encephalitis, uterine infections (causing abortion and still birth) (Papić et al. 2019), and subclinical mastitis (Constable et al. 2016).

*Listeria monocytogenes* has been extensively characterized in many animal species although farm livestock have the greatest pretentious (Chow et al. 2021). It is shed by most diseased ruminants, which are asymptomatic carriers, into their environment via their feces (Nightingale et al. 2004). It is a global environmental bacterium present in diverse farm environments, including water, silage, and feces, which are the main resources and potential reservoirs of *L. monocytogenes* in dairy farms. Consequently, the dairy cattle farms have different genotypes of *L. monocytogenes* associated with human listeriosis outbreaks (Castro et al. 2018).

The ecology of *L. monocytogenes* in the farm environment is complicated and inadequately appreciated. Furthermore, factors influencing the persistence of genotypes in dairy farms are unknown. Therefore, our knowledge about the persistence patterns and contamination methods of *L. monocytogenes* in dairy cattle farms should be improved to elucidate the contaminant source and provide crucial data for reducing transmission and developing intervention strategies against *L. monocytogenes* at the level of farms and from animals to humans (Walland et al*.* 2015; Castro et al. 2018; Chow et al. 2021).

Fecal shedding of *L. monocytogenes* has many risk factors, including inadequate hygiene, sanitation, housing conditions, silage, antibiotic therapy (approved by veterinarians), and atmospheric season (Bandelj et al. 2018). In addition, bulk tank milk (BTM), milk filter, milking machine, milk handler, fecal contamination, poor on-farm hygiene during milking, storage, and transportation are considered contamination sources (Pantoja et al. 2012).

The virulence potential of *L. monocytogenes* is determined by several molecular determinants (Camejo et al. 2011), which are classified as follows: listeriolysin O (encoded by *hlyA*), internalins (coded by *inlA*, *inlC*, and *inlJ*), virulence regulator (represented by *prfA*), actin assembly (coded by *actA*), phosphatidylinositol-phospholipase C (encoded by *plcA*), and invasion-associated protein (represented by *iap*) (Liu et al. 2007). Listeriolysin O (LLO) is a pore-forming toxin produced by *hlyA* that contributes to the lysis of bacterium-containing phagocytic vacuoles; as a result, bacterial cells are released into the host cytoplasm.

The persistent increase in antibiotic resistance among Listeria spp., notably *L. monocytogenes*, has been linked to ongoing selective pressure from widespread antibiotic usage in agriculture, animals, and humans during the last few decades (Baquero et al. 2020). *L. monocytogenes* is resistant to β-lactam, fosfomycin, third-generation cephalosporins, quinolones, erythromycin, tetracycline-minocycline, and trimethoprim (Iwu and Okoh 2020). Multi-antibiotic-resistant (MAR) *L. monocytogenes* strains are determined in food, environmental, and clinical samples (Baquero et al. 2020; Iwu and Okoh 2020; Swetha et al. 2021). Antibiotic resistance develops in these pathogens through gene mutations or acquisition of mobile genetic elements. Antibiotic resistance genes are rapidly being acquired by *L. monocytogenes*, which may have originated from commensal organisms prevalent in foods and food-processing areas. Some antibiotic resistance genes identified in *L. monocytogenes* are β-lactamase genes (*bla*SHV, *bla*CTX-M, *bla*OXA, *bla*IMP, *bla*CMY, and *bla*TEM), quinolone-resistant genes (*qnrA*, *qnrB*, *qnrS*, *gyrA*, and *parC*), macrolide-resistant genes (*erm* (A), *erm* (B), *erm*(C), *erm* (TR), *mef* (A), and *msr* (A)), and tetracycline-resistant genes (*tet*A, *tet*K, *tet*L, *tet*M, *tet*S, and *int-*Tn) (Baquero et al. 2020). However, additional information about the mechanisms of antibiotic resistance among *L. monocytogenes* strains should be obtained to design strategies that can prevent the emergence and spread of resistance and to develop innovative therapeutic approaches against multidrug-resistant organisms.

One of the critical points to avoid the distribution of healthcare-associated infections and develop infection control is distinguishing the genetic relatedness among different pathogenic strains. Repetitive element sequence-based polymerase chain reaction (PCR) is a molecular approach used to study repetitive nucleotide sequences within a bacterial genome and to cluster bacterial strains. Enterobacterial repetitive intergenic consensus (ERIC) is one of the repetitive basics characterized by the diverse patterns and numbers of bacterial genomes. *L. monocytogenes* strains possess short ERIC sequences (Jersek et al. 1999). Enterobacterial repetitive intergenic consensus polymerase chain reaction (ERIC-PCR) is a rapid, dependable, and cost-effective technique for molecular typing that distinguishes the genetic diversity among strains (Parsaie Mehr et al. 2017).

Few epidemiological surveys have been performed on *L. monocytogenes* in dairy cattle farms in Egypt and its resistance to various antibiotics. Hence, this research was designed to (a) systematically survey the prevalence and distribution of *L. monocytogenes* in three dairy cattle farms, (b) categorize the risk factors associated with *L. monocytogenes* fecal shedding in the three dairy farms, (c) determine the virulence and phenotypic and genotypic antibiotic resistance profiles of distinct types of *L. monocytogenes* strains and (d) establish the genetic diversity of *L. monocytogenes* strains through Enterobacterial repetitive intergenic consensus polymerase chain reaction (ERIC-PCR).

## Materials and methods

### Study areas

A cross-sectional study was performed to investigate the prevalence of *L. monocytogenes* and the associated risk factors in the environment of dairy cattle farms in Dakahlia Governorate, Egypt. Three dairy cattle farms were chosen on the basis of their owners’ willingness to permit recurrent sample collection. The map of Dakahlia Governorate was constructed to highlight the location of the three selected dairy cattle farms in relation to the rest of Dakahlia (Supplementary Fig. 1).

### Questionnaire preparation and data collection

For basic purposes, a structured questionnaire was prepared. The schedule was pretested with necessary adjustment to confirm the importance of the questions and the nature of the sample producers. The dairy farms were selected on the basis of owners’ willingness to allow frequent sample collection. The following data were obtained using the questionnaire: general farm information, animal movement, purchase of animals, visitors and staff, health status, and milking process, as shown in Supplementary Table 1. The farmers were asked questions, and their responses were recorded in the questionnaire. Other details, such as the sanitary condition of the farms, were collected through direct observation.

#### Assessment of production hygiene

The production hygiene of all farms was assessed depending on the premises hygiene, which was evaluated at each visit during the study period. Aspects assessed included milk room, milking station, waiting area, manure passage, resting area, cow cleanliness, feed troughs, and water troughs. Full scores of 1 to 3 were assigned for each of the evaluated sites in the three farms, where “1” referred to a major deficit in production hygiene, “2” denoted a minor deficit in production hygiene, and “3” indicated no notable deficit in production hygiene according to Castro et al. (2018). During farm visits, the production hygiene of each farm was evaluated on the basis of the cleanliness of the premises, particularly overall cleanliness, hygienic design and condition of materials, drainage, air quality, lighting, and insect control.

### Collection and processing of samples

A total of 1896 samples were collected from the three examined dairy cattle farms. Among them, 858, 432, and 606 samples were obtained from farms I, II, and III, respectively. Each farm was visited three times (in February, April, and June 2021) so that seasonal variation could be examined. At each farm level, the samples were collected from animals (milk and feces), environment (water, silage, manure, and soil), and milking equipment (teat cup swabs, milk filters, BTM samples, and floor swabs in storage areas). In farm I, 300 animal samples were obtained for each type; 15 environmental samples were acquired for each type; and 150, 30, 12, and 6 samples were obtained from teat cup swabs, milk filters, BTM samples, and floor swabs in storage areas, respectively. In farm II, 150 animal samples were collected for each type; 9 environmental samples were obtained for each type; and 75, 12, 6, and 3 samples were gathered from teat cup swabs, milk filters, BTM samples, and floor swabs in storage areas, respectively. In farm III, 210 animal samples were obtained for each type; 12 environmental samples were collected for each type; and 105, 18, 9, and 6 samples were acquired from teat cup swabs, milk filters, BTM samples, and floor swabs in storage areas, respectively.

#### Animal samples

##### A. Milk samples

Milk samples were collected before milking early in the morning in accordance with previously described methods (Scha et al. 1971). Briefly, before the samples were collected, udders, particularly teats, were cleansed and dried. Each teat end was cleaned with a pledget of cotton wet with 70% ethyl alcohol. A separate pledget of cotton was used for each teat. The first few streams of milk were discarded. About 10 ml of milk was collected into 15-ml sterile glass vials and labeled as RF (right front), and RR (right rear), LF (left front), LR (left rear).

##### B. Fecal samples

Fecal samples were obtained directly from the rectum of each cow by using a separate clean plastic sleeve for each sample. The plastic sleeves were inverted, and the content was aseptically transferred into sterile plastic vials (conical 50-ml propylene screw top; VWR International, Inc., West Chester, PA).

#### Environmental samples

##### A. Water

Water samples were collected from common water troughs in a 50-cm sterile glass bottle (American Public Health Association 1971).

##### B. Soil

Soil samples were obtained from various sites of yards, especially from the wetted region with high moisture and organic matter load at a depth of 5 cm in a sterile glass bottle fitted with a sterile glass stopper (Clegg et al. 1983).

##### C. Silage

Silage samples were gathered from silage bunkers of the three dairy farms and placed in a sterile Whirl–Pak bag (Vongkamjan et al. 2012).

##### D. Manure

Manure composite samples were aseptically obtained from various sites in each pen by using a sterile plastic sleeve. The plastic sleeve content was homogenized, and an aliquot was aseptically transferred into 50-ml plastic vials.

#### Milking equipment

Bulk tank milk samples (100 ml) were aseptically collected. Milk filter samples were obtained and aseptically transported into a sterile sealable plastic bag. For the milking equipment, a sponge was utilized to wipe the inner side of a certain area. Aseptic cotton swabs were used to collect teat cups and floor swabs in the storage area when the routine washing cycle was complete. The sponges were deposited in sterilized bags containing neutralizing buffer, and the cotton swabs were placed in aseptic tubes with 3 ml of neutralizing buffer. Each sample was loaded in coolers with ice packs and transferred to the laboratory for bacterial examination.

### Sample analysis for *L. monocytogenes*﻿

The soil, silage, fecal, and manure composite samples were weighed, diluted with 1% buffered peptone water (BPW), and pummeled (BagMixer; Weymouth, MA, Interscience Laboratories, Inc.) for bacterial analysis as formerly pronounced by Latorre et al. (2010). All samples were pre-enriched by adding 5 ml of BPW or 5 ml of milk to 5 ml of half Fraser broth (Oxoid, Basingstoke, UK) and incubated for 24 h at 37 °C. For the enrichment, 1 ml of the pre-enriched half Fraser broth was added to 9 ml of Fraser broth (Oxoid, Basingstoke, UK) for each sample and incubated at 37 °C for 24 h. Each enriched Fraser broth culture was cultured onto Palcam agar (Oxoid, Basingstoke, UK) and incubated for 48 h at 37 °C. Presumptive *L. monocytogenes* colonies (gray with black center) were biochemically identified using the following tests: catalase test, oxidase test, evaluation of hemolysis type, motility at 25 °C and 37 °C, in addition to sugar fermentation test (Van Kessel et al. 2004). The strains expressing these standard features were further tested using the API Listeria test (BioMerieux) for confirmation.

### Molecular characterization of *﻿L. monocytogenes*

The genomic DNA was extracted from overnight culture of brain heart infusion broth using the boiled lysate method (Agersborg et al. 1997). Amplification of *16S RNA* gene, virulence genes (listeriolysin O (*hlyA*), positive regulatory factor (*prfA*)), and adherence (*iap*) genes for the *L. monocytogenes* species identification was done by the standard PCR assays in Applied Biosystem, 2720 Thermal Cycler (USA), in a total volume of 25 μL consisted of 12.5 μL of 2 × PCR master mix (Promega, Madison, USA), 1 μL of individual primer (Metabion, Germany), 4.5 μL PCR-grade water, and 6 μL DNA template. The primers and PCR conditions were used as earlier described (Wang et al. 1992; Bohnert et al. 1992; Germini et al. 2009; Soni et al. 2014). The amplified PCR products were organized on a 1.5% agarose gel that was tainted by 1% ethidium bromide and photo-documented under UV illumination. *L. monocytogenes* ATCC 35,152 strain was utilized as a positive control.

### Antibiotic resistance of *L. monocytogenes*

Antibiotic-resistant *L. monocytogenes* strains were obtained via the agar disk diffusion approach on Mueller–Hinton agar (Difco), as endorsed by the Clinical and Laboratory Standards Institute (CLSI 2020). Commonly used antibiotics for humans and animals were selected. The following antimicrobial discs (Oxoid, Ltd.) were used: penicillin G (P/10 IU), amoxicillin (AML/10 μg), cloxacillin (OB/5 μg) belonging to β-lactams, cefotaxime (CTX/30 μg), cefoxitin (FOX/30 μg) belonging to cephalosporines, tetracycline (TE/30 μg) belonging to tetracyclines, streptomycin (S/10 μg), gentamicin (CN/10 μg), amikacin (AK/30 μg), neomycin (N), netiL. monocytogenesicin (NET/30 μg) belonging to aminoglycosides, chloramphenicol (C/30 μg) belonging to phenicols, ciprofloxacin (CIP/5 μg), norfloxacin (NOR/10 μg), nalidixic acid (NA/30 μg) belonging to fluoroquinolones, sulfamethoxazole/trimethoprim (SXT/25 μg) belonging to sulfonamides, vancomycin (VA/30 μg) belonging to glycopeptides, and erythromycin (E/15 μg) belonging to macrolides. *L. monocytogenes* strains were assessed as susceptible, intermediate, or resistant in accordance with the CLSI (2020) guidelines for *Staphylococcus aureus* ATCC 25,923 and *Escherichia coli* ATCC 25,922. The strains displaying resistance to at least three classes of the antimicrobial agents tested were considered MAR strains. The MAR index of each resistant pattern was calculated using the formula provided by Singh et al. (2010). MAR index = number of resistance (strains classified as intermediate based on inhibition zone were considered as sensitive for MAR index) antibiotics/total number of antibiotics tested.

### Molecular detection of antibiotic-resistant genes

The resistant *L. monocytogenes* strains were investigated in terms of the following resistance genes through simplex polymerase chain reaction: *bla*CTX-M, *bla*DHA-1, and *bla*SFO-1 for extended-spectrum β-lactamases (ESBL); *qnrA*, *qnrB*, *qnrS*, *gyrA*, and *parC* for quinolones; *erm* (A), *erm* (B), *erm* (C), *erm* (TR), *mef* (A), and *msr* (A) for macrolides; *dfrD* for trimethoprim; and *tet* (K), *tet* (L), *tet* (M), *tet* (S), and *int-*Tn, which encodes the Tn*916-*Tn*1545* integrase of the transposon family, for tetracyclines (Morvan et al. 2010). The primer sequence, cycling conditions, and expected amplicon size are illustrated in Supplementary Table 2. As previously stated, PCR and electrophoresis were performed.

### Genetic diversity analysis using ERIC-PCR

The *L. monocytogenes* strains were genotyped via the enterobacterial repetitive intergenic consensus polymerase chain reaction (ERIC-PCR) fingerprinting assay as described in previous study (Bilung et al. 2018). Genomic DNA was extracted using a QIAamp DNA Mini Kit (Qiagen, Germany). The oligonucleotide forward primer sequence was ERIC1 primer 5′ATGTAAGCTCCTGGGGATTCAC-3′, and the reverse primer sequence was ERIC1 primer 5′ AAGTAAGTGACTGGGGTGAGCG-3′ (Versalovic et al. 1991). Each 25 μL of the PCR mixture was composed of 12.5 μL of 2 × PCR master mix (Promega, Madison, USA), 1 μL of individual primer (Metabion, Germany), 4.5 μL of PCR-grade water, and 6 μL of DNA template. PCR amplification was performed with the following thermal cycles (Biometra): primary denaturation at 94 °C for 5 min; 35 cycles of denaturation at 94 °C for 30 s, annealing at 52 °C for 1 min, and extension at 72 °C for 1 min; and a final cycle of 72 °C for 12 min. The amplified PCR products were resolved by electrophoresis on 1.5% agarose gels and photo-documented under UV illumination (Alpha Innotech). ERIC fingerprinting data were converted into a binary code based on the presence or deficiency of each band. Dendrogram was created by the unweighted pair group approach with arithmetic average (UPGMA) and Ward’s hierarchical clustering routine. Cluster analysis and dendrogram construction were presented with SPSS, version 22 (IBM 2013) (Hunter 1990). Similarity index (Jaccard/Tanimoto coefficient and number of intersecting elements) among all samples was analyzed by the online tool (https://planetcalc.com/1664/).

### Statistical analysis

Data was recorded using Microsoft Excel spreadsheet (version 15.0), and the investigation was conducted using SPSS (Statistical Set for Social Science) software version 22. Accordingly, descriptive statistics such as percentages and frequency distribution were utilized to determine the prevalence. The antibiotic sensitivity patterns were presented in percentages. Probability level (*P*) was calculated by using non-parametric test (Chi-square test).

## Results

### Prevalence and ecology of *L. monocytogenes﻿*﻿ in the dairy cattle farms

Of 1896 samples that were collected, 7.23% (farm I, 3.4%; farm II, 15.2%; and farm III, 6.9%) were positive for *L. monocytogenes* (Table 1). *L. monocytogenes* (7.23%) was more predominant in animal samples (32.1%) than in milking equipment (21.9%) and environmental samples (16.8%). *L. monocytogenes* was determined in all the sampled areas of the three investigated farms except water and floor swabs from the storage area of farm I which were *L. monocytogenes* negative. Overall, *L. monocytogenes* was more prevalent in farm I (for all types of samples) than in farms II and III. The prevalence of *L. monocytogenes* in fecal samples was slightly higher than that in milk samples and varied among the three farms (I, 3% and 2.7%; II, 14.7% and 12%; III, 6.7% and 6.2%, respectively). In environmental samples, *L. monocytogenes* was found most commonly in silage (farm I, 20%; farm II, 44.4%; and farm III, 25%) followed by manure (farm I, 13.3%; farm II, 33.3%; farm III, 16.6%), water (farm II, 22.2%, and farm III, 8.3%) and soil (farm I, 6.7%; farm II, 11.1%; and farm III, 8.3%). In milking equipment, the prevalence of *L. monocytogenes* was the highest in teat cup swabs (farm I, 8.3%; farm II, 50%; farm III, 22.2%) and floor swabs in the storage area (farm I, 0%; farm II, 66.7%; and farm III, 16.7%) followed by milk filters (farm I, 3.3%; farm II, 16.7%; and farm III, 5.6%) and BTM samples (farm I, 2.7%; farm II, 12%; and farm III, 3.8%).

**Table 1:** Prevalence and distribution of *Listeria monocytogenes* in the three dairy cattle farms

Samples	Farm I		Farm II		Farm III		Total no. of positive (%)
	**Total no. of samples**	**Isolate no. (%)**	**Total no. of samples**	**Isolate no. (%)**	**Total no. of samples**	**Isolate no. (%)**	
**Animal samples**							
Fecal samples	300	9 (3)	150	22 (14.7)	210	14 (6.7)	45 (6.8)
Milk samples	300	8 (2.7)	150	18 (12)	210	13 (6.2)	39 (5.9)
**Environmental samples**							
Water	15	0	9	2 (22.2)	12	1 (8.3)	3 (8.3)
Silage	15	3 (20)	9	4 (44.4)	12	3 (25)	10 (27.8)
Manure	15	2 (13.3)	9	3 (33.3)	12	2 (16.6)	7 (19.4)
Soil	15	1(6.7)	9	1 (11.1)	12	1 (8.3)	3 (8.3)
**Milking equipment**							
Bulk tank milk samples	150	4 (2.7)	75	9 (12)	105	4 (3.8)	17 (5.2)
Milk filters	30	1 (3.3)	12	2 (16.7)	18	1 (5.6)	4 (6.7)
Teat cups swabs	12	1 (8.3)	6	3 (50)	9	2 (22.2)	6 (22.2)
Floor swabs in the storage area	6	0	3	2 (66.7)	6	1 (16.7)	3 (20)
Total	858	29 (3.4)	432	66 (15.2)	606	42 (6.9)	137 (7.3)

### Associated risk factors of the fecal shedding of *L. monocytogenes*﻿

The prevalence of *L. monocytogenes* showed seasonal variation in different samples collected from the animals, environment, and milking equipment of the three dairy cattle farms (Table 2). Analyses revealed that *L. monocytogenes* was significantly (*P* = 0.000) more prevalent in all samples obtained in winter (farm I, 6.6%; farm II, 25.7%; and farm III, 13.7%) than in spring (farm I, 3%; farm II, 13.9%; and farm III, 6.8%) and summer (farm I, 0.35%; farm II, 6.3%; farm III, 0%). The seasonal prevalence of *L. monocytogenes* in all sample categories for each farm was significantly higher (*P* = 0.043–0.000) in winter than in spring and summer. In winter, the prevalence of *L. monocytogenes* was the highest in the environmental samples followed by the milking equipment. Conversely, the lowest prevalence was observed in the animal samples, on the level of three farms.

**Table 2:** Seasonal variation in the prevalence of *Listeria monocytogenes* in the three dairy cattle farms

Samples		Season						*P* value
		Winter		Spring		Summer		
		Total no. of samples	No. of positive samples (%)	Total no. of samples	No. of Positive samples (%)	Total no. of samples	No. of Positive samples (%)	
Farm I	Animal samples	20	9 (4.5)	200	7 (3.5)	200	1 (0.5)	0.043
	Environmental samples	20	5 (25)	20	1 (5)	20	0	0.018
	Milking equipment	6	5 (7.6)	66	1 (1.5)	66	0	0.027
Total		286	**19 (6.6)**	286	9 (3)	286	1 (0.35)	0.000**
Farm II	Animal samples	100	22 (22)	100	12 (12)	100	6 (6)	0.004
	Environmental samples	12	6 (50)	12	2 (16.7)	12	2 (16.7)	0.1
	Milking equipment	32	9 (28.1)	32	6 (18.8)	32	1 (3.1)	0.025
Total		144	**37 (25.7)**	144	20 (13.9)	**144**	**9 (6.3)**	0.000**
Farm III	Animal samples	140	17 (12.1)	140	10 (7.1)	140	0	0.000
	Environmental samples	16	6 (37.5)	16	1 (6.3)	16	0	0.006
	Milking equipment	49	5 (10.2)	49	3 (6.1)	49	0	0.016
Total		**205**	**28 (13.7)**	205	**14 (6.8)**	**205**	**0**	0.000**

^**^Superscripts indicate highly significant difference for seasonal prevalence of *L. monocytogenes* across all sample categories within each farm at *p* ≤ 0.05

The three farms had different hygiene levels (Table 3). The overall hygiene scores (2.8 to 2.5) of farms I and III were higher than that of farm II (1.4). The rank of each farm according to hygiene score had a strong inverse connection with the prevalence of *L. monocytogenes*. Differences in production hygiene stated in the questionnaire might be due to the considerably greater incidence of *L. monocytogenes* in farm II (15.2%) than in farms I (6.9%) and III (3.4%). The fecal shedding of *L. monocytogenes* was positively associated (*P* < 0.05) with age, parity, and milk yield (Table 4). At the farm level, the higher levels of *L. monocytogenes* shedding in the feces of dairy cattle were associated with increasing age of > 8 years (farm I, 12%; farm II, 27.8%; and farm III, 17.4%), number of parities of > 4 (farm I, 6.9%; farm II, 29.3%; and farm III, 33.3%), and milk yield of > 18 kg/day (farm I, 4%; farm II, 40%; and farm III, 14.3%).

**Table 3:** Evaluation of hygiene on the investigated dairy cattle farms

Area	Hygiene score by farm								
	Farm I			Farm II			Farm III		
	Winter	Spring	Summer	Winter	Spring	Summer	Winter	Spring	Summer
Milk room	3	3	3	2	2	2	2	3	3
Milking station *a*	3	3	3	1	1	1	2	2	2
Waiting area	3	3	3	2	2	2	3	3	3
Manure passage	2	2	3	1	1	1	2	2	3
Resting area	2	3	3	2	1	2	2	2	3
Cow cleanliness	2	3	3	1	1	1	2	2	2
Feed troughs	2	3	3	1	1	2	2	2	3
Water troughs	3	3	3	1	1	2	3	3	3
Mean score of evaluation overall	2.5	2.9	3	1.4	1.3	1.6	2.3	2.4	2.8
Mean hygiene score	2.8			1.4			2.5		

*a*: Refers to the milking parlor in farms I and III or the milking unit of an automatic milking system in farm II

Each area received a score from 1 to 3, where 1 is major deficits in production hygiene, 2 is minor deficits in production hygiene, and 3 is no notable deficits in production hygiene

**Table 4:** Animal’s risk factors related to shedding of *Listeria monocytogenes* in feces on the level of three dairy cattle farms

Risk factor	Farm I		Farm II		Farm III	
	Total no. of samples	Positive no. (%)	Total no. of samples	Positive no. (%)	Total no. of samples	Positive no. (%)
**Age (years)**						
3–5	136	0	62	2 (3.2)	110	4 (3.6)
6–8	89	0	34	5 (14.7)	77	6 (7.8)
> 8	75	9 (12)	54	15 (27.8)	23	4 (17.4)
***P* value**		0.000		0.001		0.05
**Parity (no.)**						
Up to 2	18	0	49	2 (4)	69	2 (2.9)
2–4	180	2 (1.1)	60	8 (13.3)	120	5 (4.2)
> 4	102	7 (6.9)	41	12 (29.3)	21	7 (33.3)
***P* value**		0.01		0.003		0.000
**Milk yield (kg/day)**						
Low (< 12)	0	0	50	2 (4)	8	0
Medium (12–18)	80	0	70	8 (11.4)	114	0
High (over 18)	220	9 (4)	30	12 (40)	98	14 (14.3)
***P* value**		0.000		0.000		0.000

Factors statistically significant at *p* ≤ 0.05

### Virulence genes in *L. monocytogenes﻿*﻿ strains

All strains (*n* = 137) from various farm samples were examined for the existence of virulence genes, as demonstrated in Table 5. A total of 93 *L. monocytogenes* strains were positive for both *hlyA* and *prfA* genes, i.e., 56 animal strains (60.2%), 22 milking equipment strains (23.7%), and 15 environmental strains (16.1%). The 44 remaining strains carried only one virulence gene: 30 strains with *hly*A and 14 strains with *prfA*. Conversely, none of the strains had *iap*.

**Table 5:** Ecological distribution of antibiotic resistance of *Listeria monocytogenes* strains in the three examined dairy cattle farms (*n* = 137)

Samples		Total no. of isolates	Antibiotics																	
			P	N	FOX	NA	AML	OB	CTX	AK	E	NOR	TE	CN	S	SXT	CIP	C	NET	VA
Farm I	Animal samples	17	17	17	17	17	13	11	9	5	5	0	0	9	4	0	0	0	0	0
	Environmental samples	6	6	6	6	4	4	3	5	3	3	0	0	1	2	0	0	0	0	0
	Milking equipment	6	6	6	6	4	4	3	2	2	2	0	0	1	2	0	0	0	0	0
	Total	29	29	29	29	21	21	17	16	10	10	0	0	11	8	0	0	0	0	0
Farm II	Animal samples	40	40	40	40	40	40	40	40	40	40	36	29	31	30	25	17	0	0	0
	Environmental samples	10	10	10	10	10	10	10	10	10	10	7	6	6	8	5	8	0	0	0
	Milking equipment	16	16	16	16	16	16	16	16	16	16	12	11	10	10	7	7	0	0	0
	Total	66	66	66	66	66	66	66	66	66	66	55	46	47	48	37	32	0	0	0
Farm III	Animal samples	27	27	27	27	27	27	27	21	23	23	21	17	9	10	19	16	5	0	0
	Environmental samples	7	7	7	7	7	7	7	5	6	6	5	7	1	2	4	4	1	0	0
	Milking equipment	8	8	8	8	8	8	8	6	8	8	6	3	5	3	7	5	3	0	0
	Total	42	42	42	42	42	42	42	32	37	37	32	27	15	15	30	25	9	0	0
Total		137	137	137	137	137	129	125	114	113	113	87	73	73	71	67	57	9	0	0
Resistance (%)		100	100	100	100	100	94.2	91.2	83.2	82.5	82.5	62.8	53.3	53.3	50.4	48.9	32	6.6	0	0

*P*, penicillin; *N*, neomycin; *FOX*, cefoxitin; *NA*, nalidixic acid; *AML*, amoxicillin; *OB*, cloxacillin; *CTX*, cefotaxime; *AK*, amikacin; *E*, erythromycin; *NOR*, norfloxacin; *TE*, tetracycline; *CN*, gentamicin; *S*, streptomycin; *SXT*, sulphamethazole/trimethoprim; *CIP*, ciprofloxacin; *C*, chloramphenicol; *NET*, netilmicin; VA, vancomycin

### Antibiotic resistance of *Listeria monocytogenes*﻿ strains

The antibiotic susceptibility test results of 137 *L. monocytogenes* strains screened for 18 relevant antimicrobial agents which belong to eight various classes of antibiotics including beta-lactams, tetracyclines, quinolones, phenicols, sulfonamides, macrolides, aminoglycoside, and glycopeptides are illustrated in Table 6. The highest resistance of the *L. monocytogenes* strains was observed in penicillin, neomycin, cefoxitin, and nalidixic acid (100%), followed by amoxicillin (94.2%), cloxacillin (91.2%), cefotaxime (83.2%), amikacin (82.5%), erythromycin (82.5%), and norfloxacin (62.8%). By contrast, the resistance of the *L. monocytogenes* strains to tetracycline, gentamicin, streptomycin, and sulfamethoxazole-trimethoprim was low (53.3%, 53.3%, 50.4%, and 48.9%, respectively). According to sample type, the strains from the animal samples were highly resistant to amoxicillin (95.2%, 80/84) and cloxacillin (92.9%, 78/84). The strains from the environmental samples were also highly resistant to cefotaxime (86.95%, 20/23).

**Table 6:** ERIC-PCR, antibiotic resistance and virulence of *Listeria monocytogenes* isolated from dairy cattle farms (*n* = 137)

Farm	Source	Type of sample	Isolates no. or ID	ERIC-PCR Type	*Antibiotics pattern	Virulence genes	Antibiotics resistance genes
**Farm I**	Animals	Milk	14	F	4	*prfA*	CTX-M
			21	F	8	*prfA*	CTX-M, *dfrD*
			25	K	9	*hlyA*, *prfA*	CTX-M, DHA, *qnrA*, *qnrB*, *qnrS*, *parC*, *ermB*, *msrA*
			15	L	4	*hlyA*	DHA, *qnrS*, *dfrD*
			24	M	9	*hlyA*,*prfA*	CTX-M, *qnrA*, *qnrS*, *parC*, *ermB*, *msrA*, *dfrD*
			1	Q	1	*prfA*	DHA, *qnrS*, *dfrD*
			19	Q	5	*hlyA*	CTX-M, *dfrD*
			22	T	14	*hlyA*, *prfA*	CTX-M, DHA, *qnrA*, *qnrB*, *qnrS*, *parC*, *ermB*, *msrA*
		Feces	11	E	3	*hlyA*	DHA, *qnrS*, *dfrD*
			17	I	4	*hlyA*	DHA, *qnrS*
			5,6	F	2	*hlyA*	CTX-M, DHA, *qnrS*
			9	F	3	*hlyA*,	DHA, *qnrS*
			2	G	1	*hlyA*, *prfA*	DHA, *qnrS*, *dfrD*
			18	G	5	*prfA*	CTX-M, *dfrD*
			23	G	14	*hlyA*	CTX-M, *qnrA*, *qnrB*, *qnrS*, *parC*, *ermB*, *dfrD*
			13	L	4	*hlyA*	DHA, *qnrS*, *dfrD*
	Environment	Silage	20	G	8	*hlyA*	CTX-M, *qnrS*, *ermB*, *dfrD*
			12	F	3	*hlyA*	CTX-M
			29	J	9	*prfA*	CTX-M, *qnrA*, *qnrB*, *qnrS*, *parC*, *ermB*
		Manure	8	J	2	*prfA*	CTX-M, *qnrS*, *dfrD*
			26	M	9	*hlyA*, *prfA*	CTX-M, *qnrA*, *qnrS*, *parC*, *ermB*, *dfrD*
		Soil	7	N	2	*hlyA*	CTX-M, *qnrS*, *dfrD*
	Milking equipment	BTM	16	A	4	*hlyA*	CTX-M, *qnrS*
			10	E	3	*hlyA*	CTX-M, *qnrS*, *dfrD*
			4	F	1	*prfA*	*qnrS*
			27	M	9	*hlyA*, *prfA*	CTX-M, *qnrA*, *qnrS*,*parC*, *ermB*, *dfrD*
		Teat cups swab	28	S	9	*hlyA*	CTX-M, *qnrA*, *qnrB*,*qnrS*, *ermB*, *msrA*, *parC*
		Milk filter	3	Q	1	*prfA*	DHA, *qnrS*, *dfrD*
**Farm II**	Animals	Milk	70	A	11	*hlyA*, *prfA*	CTX-M, *qnrA*, *qnrB*,*qnrS*, *parC*, *ermB*, *msrA*, *dfrD*
			76	A	12	*hlyA*, *prfA*	CTX-M, *qnrA*, *qnrB*, *qnrS*, *parC*, *ermB*, *msrA*, *dfrD*
			40,56	G	24	*hlyA*, *prfA*	CTX-M, DHA, *qnrA*, *qnrB*, *qnrS*, *parC*, *ermB*, *msrA*, *dfrD*, *tetM*, *int-Tn*
			88	K	7	*hlyA*, *prfA*	CTX-M, *qnrA*, *qnrB*, *qnrS*, *parC*, *ermB*, *msrA*, *dfrD*
			80	L	12	*hlyA*, *prfA*	CTX-M, DHA, *qnrA*, *qnrS*, *parC*, *ermB*, *msrA*
			35	M	20	*hlyA*, *prfA*	CTX-M, DHA, *qnrS*, *ermB*, *msrA*, *dfrD*, *tetM*, *int-Tn*
			59	M	24	*prfA*	CTX-M, DHA, *qnrA*, *qnrB*, *qnrS*, *parC*, *ermB*, *msrA*, *dfrD*, *tetM*, *int-Tn*
			51	N	25	*hlyA*, *prfA*	CTX-M, DHA, *qnrA*, *qnrB*, *qnrS*, *parC*, *ermB*, *msrA*, *dfrD*, *tetM*
			39	Q	20	*hlyA*, *prfA*	CTX-M, DHA, *qnrA*, *qnrS*, *parC*, *ermB*, *dfrD*, *tetM*, *int-Tn*
			41	Q	18	*hlyA*, *prfA*	CTX-M, DHA, *qnrA*, *qnrS*, *parC*, *ermB*, *dfrD*, *tetM*
			45	Q	25	*hlyA*, *prfA*	CTX-M, DHA, *qnrA*, *qnrS*, *parC*, *ermB*, *dfrD*, *tetM*, *int-Tn*
			65	Q	19	*hlyA*, *prfA*	CTX-M, DHA, *qnrA*, *qnrB*, *qnrS*, *parC*, *ermB*, *dfrD*, *tetM*
			66	S	19	*hlyA*, *prfA*	CTX-M, DHA, *qnrA*, *qnrB*, *qnrS*, *parC*, *ermB*, *tetM*
			34	T	20	*prfA*	CTX-M, DHA, *qnrA*, *qnrS*, *parC*, *ermB*, *dfrD*, *tetM*
			47	T	25	*hlyA*, *prfA*	CTX-M, DHA, *qnrA*, *qnrS*, *parC*, *ermB*, *dfrD*, *tetM*
			38	U	20	*hlyA*, *prfA*	CTX-M, DHA, *dfrD*, *tetM*
			44	U	25	*hlyA*, *prfA*	CTX-M, DHA, *qnrA*, *qnrB*, *qnrS*, *parC*, *ermB*, *msrA*, *dfrD*, *tetM*
		Feces	91, 93	A	15	*hlyA*, *prfA*	CTX-M, DHA, *qnrS*, *ermB*
			94, 95	A	24	*hlyA*, *prfA*	CTX-M, DHA, *qnrA*, *qnrB*, *qnrS*, *parC*, *ermB*, *msrA*, *dfrD*, *tetM*, *int-Tn*
			32	A	24	*hlyA*, *prfA*	CTX-M, *qnrA*, *qnrB*, *qnrS*, *parC*, *ermB*, *dfrD*, *tetM*, *int-Tn*
			89	C	7	*hlyA*, *prfA*	CTX-M, DHA, *qnrA*, *qnrB*, *qnrS*, *parC*, *ermB*, *dfrD*
			92	C	15	*hlyA*, *prfA*	CTX-M, DHA, *qnrS*, *ermB*, *dfrD*
			64	C	24	*hlyA*, *prfA*	CTX-M, *qnrA*, *qnrB*, *qnrS*, *parC*, *ermB*, *msrA*, *dfrD*, *tetM*, *int-Tn*
			30	F	9	*hlyA*, *prfA*	CTX-M, *qnrS*, *ermB*
			31,67,68	F	24	*hlyA*, *prfA*	CTX-M, *qnrA*, *qnrB*, *qnrS*, *parC*, *ermB*, *msrA*, *dfrD*, *tetM*, *int-Tn*
			52	F	23	*hlyA*	CTX-M, *tetM*, *int-Tn*
			79	I	16	*hlyA*, *prfA*	CTX-M, DHA, qnrS, ermB, msrA, dfrD, tetM, int-Tn
			81	I	12	*hlyA*, *prfA*	CTX-M, *qnrS*
			90	I	15	*hlyA*, *prfA*	CTX-M, DHA, *qnrS*, *ermB*, *dfrD*
			33	J	24	*hlyA*	CTX-M, DHA, *qnrA*, *qnrB*, *qnrS*, *parC*, *ermB*, *msrA*, *dfrD*, *tetM*, *int-Tn*
			43,49,50	J	25	*hlyA*, *prfA*	CTX-M, DHA, *qnrA*, *qnrB*, *qnrS*, *parC*, *ermB*, *msrA*, *dfrD*, *tetM*, *int-Tn*
			58	G	24	*hlyA*, *prfA*	CTX-M, DHA, *qnrA*, *qnrB*, *qnrS*, *parC*, *ermB*, *msrA*, *dfrD*, *tetM*, *int-Tn*
			57	Q	24	*hlyA*, *prfA*	CTX-M, *qnrA*, *qnrB*, *qnrS*, *parC*, *ermB*, *dfrD*, *tetM*
	Environment	Water	84	I	12	*hlyA*, *prfA*	CTX-M, *qnrS*
			53	J	23	*hlyA*, *prfA*	*qnrS*, *ermB*, *dfrD*, *tetM*, *int-Tn*
		Silage	54	A	23	*hlyA*, *prfA*	CTX-M, *qnrA*, *qnrB*, *qnrS*, *parC*, *ermB*, *tetM*, *int-Tn*
			61	C	24	*hlyA*, *prfA*	CTX-M, *qnrA*, *qnrB*, *qnrS*, *parC*, *ermB*, *dfrD*, *tetM*, *int-Tn*
			82	E	17	*hlyA*, *prfA*	CTX-M, *qnrA*, *qnrB*, *qnrS*, *parC*, *ermB*, *dfrD*, *tetM*
			71	F	11	*hlyA*, *prfA*	CTX-M, *qnrA*, *qnrB*, *qnrS*, *parC*, *ermB*
		Manure	75	M	11	*hlyA*, *prfA*	CTX-M, DHA, *qnrA*, *qnrB*, *qnrS*, *parC*, *ermB*
			86	M	7	*hlyA*, *prfA*	CTX-M, *qnrA*, *qnrB*, *qnrS*, *parC*, *ermB*, *dfrD*
			55	Q	23	*prfA*	CTX-M, *qnrA*, *qnrB*, *qnrS*, *parC*, *ermB*, *dfrD*, *tetS*
		Soil	62	M	24	*hlyA*, *prfA*	CTX-M, *qnrA*, *qnrB*, *qnrS*, *parC*
	Milking equipment	BTM	77,78	L	16	*hlyA*, *prfA*	CTX-M, DHA, *dfrD*, *tetM*, *int-Tn*
			36	M	20	*hlyA*, *prfA*	CTX-M, DHA, *qnrS*, *ermB*, *msrA*, *dfrD*, *tetM*, *int-Tn*
			60	M	21	*hlyA*, *prfA*	CTX-M, *qnrA*, *qnrB*, *qnrS*, *parC*, *ermB*, *tetM*
			73,74	M	11	*hlyA*, *prfA*	CTX-M, DHA, *qnrA*, *qnrB*, *qnrS*, *parC*, *ermB*
			42	Q	25	*hlyA*, *prfA*	CTX-M, DHA, *qnrA*, *qnrS*, *parC*, *ermB*, *msrA*, *dfrD*, *tetM*, *int-Tn*
			63	I	24	*hlyA*, *prfA*	CTX-M, *qnrA*, *qnrB*, *qnrS*, *parC*, *ermB*, *msrA*, *dfrD*, *tetM*, *int-Tn*
			37	J	20	*hlyA*, *prfA*	CTX-M, *dfrD*, *tetM*
		Teat cups swab	48	E	25	*hlyA*, *prfA*	CTX-M, *dfrD*, *tetM*
			46	J	25	*hlyA*, *prfA*	CTX-M, DHA, *qnrA*, *qnrB*, *qnrS*, *parC*, *ermB*, *msrA*, *dfrD*, *tetM*, *int-Tn*
			83	K	17	*hlyA*, *prfA*	CTX-M, DHA, *qnrS*, *ermB*, *msrA*, *dfrD*, *tetM*, *int-Tn*
		Milk filter	72	S	11	*hlyA*, *prfA*	CTX-M, DHA, *qnrA*, *qnrB*, *qnrS*, *parC*, *ermB*
			69	T	24	*hlyA*, *prfA*	CTX-M, DHA, *qnrA*, *qnrB*, *qnrS*, *parC*, *ermB*, *msrA*, *dfrD*, *tetM*, *int-Tn*
		Floor swabs	85	H	12	*hlyA*, *prfA*	CTX-M, *qnrS*, *ermB*, *dfrD*
			87	K	7	*hlyA*, *prfA*	CTX-M, *qnrA*, *qnrB*, *qnrS*, *parC*, *ermB*, *dfrD*
**Farm III**	Animals	Milk	115,116	C	21	*hlyA*, *prfA*	CTX-M, DHA, *qnrA*, *qnrB*, *qnrS*, *parC*, *ermB*, *tetS*
			96,97,98	L	24	*hlyA*	CTX-M, DHA, *qnrA*, *qnrB*, *qnrS*, *parC*, *ermB*
			135,136	O	13	*hlyA*, *prfA*	CTX-M, DHA, *qnrS*, *ermB*, *msrA*, *tetM*, *int-Tn*
			101	P	24	*hlyA*	CTX-M, DHA, *qnrA*, *qnrB*, *qnrS*, *parC*, *ermB*, *dfrD*, *tetM*
			102,103	R	22	*hlyA*	CTX-M, DHA, *qnrS*, *ermB*, *msrA*, *dfrD*, *tetM*, *int-Tn*
			137	T	6	*hlyA.prfA*	CTX-M, DHA, *qnrA*, *qnrB*, *qnrS*, *parC*, *ermB*, *dfrD*
			128	V	10	*hlyA*, *prfA*	CTX-M, DHA, *qnrS*, *ermB*, *dfrD*, *tetM*
			127	E	6	*hlyA*, *prfA*	CTX-M, DHA, *qnrA*, *qnrB*, *qnrS*, *parC*, *ermB*
		Feces	131	B	6	*PrfA*	CTX-M, DHA, *qnrA*, *qnrB*, *qnrS*, *parC*, *ermB*
			124,125	C	10	*hlyA*, *PrfA*	CTX-M, DHA, *qnrS*, *ermB*, *dfrD*
			106, 107, 109	D	22	*hlyA*	CTX-M, DHA, *qnrS*, *ermB*, *dfrD*, *tetS*
			111, 112, 113, 114	E	21	*hlyA*, *prfA*	CTX-M, DHA, *qnrA*, *qnrB*, *qnrS*, *parC*, *ermB*, *tetM*
			120, 122, 123	E	6	*hlyA*, *prfA*	CTX-M, DHA, *qnrA*, *qnrB*, *qnrS*, *parC*, *ermB*, *dfrD*
			129	V	10	*prfA*	CTX-M, DHA, *dfrD*
	Environment	Water	104	D	22	*hlyA*	CTX-M, DHA, *qnrS*, *ermB*, *dfrD*, *tetS*
		Silage	134	B	13	*hlyA*, *prfA*	CTX-M, *qnrS*, *ermC*, *tetS*
			118	C	21	*hlyA*, *prfA*	CTX-M, DHA, *qnrA*, *qnrB*, *qnrS*, *parC*, *ermC*, *tetS*
			119	E	21	*hlyA*, *prfA*	CTX-M, DHA, *qnrA*, *qnrB*, *qnrS*, *parC*, *ermB*, *tetM*
		Manure	130	V	6	*hlyA*, *prfA*	CTX-M, DHA, *qnrA*, *qnrB*, *qnrS*, *parC*, *ermB*
			126	V	10	*hlyA*, *prfA*	CTX-M, DHA, *qnrS*, *ermB*, *dfrD*, *tetM*
		Soil	133	S	6	*prfA*	CTX-M, *qnrA*, *qnrB*, *qnrS*, *parC*, *ermB*, *dfrD*
	Milking equipment	BTM	99	L	24	*hlyA*	CTX-M, DHA, *qnrA*, *qnrB*, *qnrS*, *parC*, *ermB*
			108	P	22	*hlyA*, *prfA*	CTX-M, DHA, *qnrS*, *ermB*, *dfrD*, *tetS*
			105	R	22	*hlyA*	CTX-M, DHA, *qnrS*, *ermB*, *msrA*, *dfrD*, *tetM*, *int-Tn*
			117	T	21	*hlyA*, *prfA*	CTX-M, DHA, *qnrA*, *qnrB*, *qnrS*, *parC*, *ermB*, *dfrD*, *tetS*
		Teat cup swab	100	I	24	*hlyA*	CTX-M, DHA, *qnrA*, *qnrB*, *qnrS*, *parC*, *ermB*, *dfrD*
			110	P	22	*hlyA*, *prfA*	CTX-M, DHA, *qnrS*, *ermB*, *dfrD*, *tetS*
		Milk filter	121	E	6	*hlyA*, *prfA*	CTX-M, DHA, *qnrA*, *qnrB*, *qnrS*, *parC*, *ermB*, *dfrD*
		Floor swab	132	V	6	*hlyA*, *prfA*	CTX-M, DHA, *qnrA*, *qnrB*, *qnrS*, *parC*, *ermB*

^*^Antibiotic patterns: **1**;P, N, FOX, NA; **2**;P, N, FOX, NA, CTX; **3**;P, N, FOX, NA, AML; **4**;P, N, FOX, NA, AML, OB, CN; **5**;P, N, FOX, NA, AML, OB, CTX, CN; **6**;P, N, FOX, NA, OB, AK, SXT, CIP; **7**;P, N, FOX, NA, OB, CTX, AK, CIP; **8**;P, N, FOX, NA, AML, OB, CTX, AK, CN; **9**; P, N, FOX, NA, AML, OB, CTX, AK, S; **10**; P, N, FOX, NA, AML, OB, CTX, NOR, SXT; **11**; P, N, FOX, NA, OB, CTX, AK, S, CIP; **12**;P, N, FOX, NA, OB, CTX, NOR, AK, CN; **13**; P, N, FOX, NA, OB, CTX, NOR, AK, TE; **14**; P, N, FOX, NA, AML, OB, CTX, AK, CN, S; **15**; P, N, FOX, NA, AML, OB, CTX, NOR, AK, S; **16**; P, N, FOX, NA, OB, CTX, NOR, AK, TE, CN; **17**; P, N, FOX, NA, OB, CTX, NOR, AK, TE, S; **18**;P, N, FOX, NA, AML, OB, CTX, NOR, AK, TE, S; **19**;P, N, FOX, NA, OB, CTX, NOR, AK, TE, CN, S; **20**; P, N, FOX, NA, OB, CTX, NOR, AK, TE, CN, SXT; **21**; P, N, FOX, NA, AML, OB, CTX, NOR, AK, TE, S, CIP; **22**;P, N, FOX, NA, OB, CTX, NOR, AK, TE, CN, SXT, C; **23**;P, N, FOX, NA, OB, CTX, NOR, AK, TE, CN, S, CIP; **24**; P, N, FOX, NA, OB, CTX, NOR, AK, TE, CN, SXT, S, CIP; **25**;P, N, FOX, NA, OB, CTX, NOR, AK, TE, CN, SXT, S

*Listeria monocytogenes* strains showed 25 multi-resistance patterns to antibiotics ranging from 4 to 14 (Supplementary Table 3). The predominant multi-resistance pattern was P, N, FOX, NA, OB, CTX, NOR, AK, E, TE, CN, SXT, S, CIP (16.8%, 23/137). This pattern was followed by P, N, FOX, NA, OB, AK, E, SXT, CIP; P, N, FOX, NA, AML, OB, CTX, NOR, AK, E, TE, S, CIP and P, N, FOX, NA, OB, CTX, NOR, AK, E, TE, CN, SXT, S (7.3%, 10/137) for each. These four resistance patterns were found in 38.7% (53/137) of all strains. As for the strains from the farms, the dominant multi-resistance patterns from farm I strains were P, N, FOX, NA, AML, OB, CN (20.7%, 6/29) and P, N, FOX, NA, AML, OB, CTX, AK, E, S (17.2%, 5/29). The dominant patterns of the strains from farm II were P, N, FOX, NA, OB, CTX, NOR, AK, E, TE, CN, SXT, S, CIP (25.8%, 17/66) and P, N, FOX, NA, OB, CTX, NOR, AK, E, TE, CN, SXT, S (15.2%, 10/66). The most common pattern of the strains from farm III was P, N, FOX, NA, OB, AK, E, SXT, CIP (23.8%, 10/42).

All *L. monocytogenes* strains (100%) had MAR to at least 4 of the 18 antibiotics examined. Their MAR index ranged from 0.22 to 0.78. The highest MAR index of 0.78 was recorded in 24.1% (33/137) isolates from which 27 strains were found in farm II. Furthermore, a MAR index of 0.72 was detected in 16.8% (23/137) of isolates.

### Antibiotic resistance genes

The PCR screening of the antibiotic resistance genes in MAR *L. monocytogenes* strains showed that all examined strains (*n* = 137) contained at least one antibiotic resistance gene (Table 5). In particular, 127 (92.7%) *L. monocytogenes* strains had *bla*CTX-M gene, and only 91 (66.4%) strains were positive for *bla*DHA-1 resistance gene. Conversely, no strain had *bla*SFO-1 gene. Genotyping analysis revealed the existence of quinolone resistance genes: *qnrS* in 125 (91.2%) strains, *qnrA* and *parC* in 80 (58.4%) strains, and *qnrB* in 70 (51%) strains. By contrast, no strain had *gyrA* gene. Macrolide resistance genes were also detected: *erm* (B) (76.6%, 105/137), *erm* (C) (1.5%, 2/137), and *msr* (A) genes (27%, 37/137). However, *erm* (A), *erm* (TR), and *mef* (A) genes were not present in the examined strains. trimethoprim *dfrD* gene was greatly abundant in 65.7% (*n* = 90) of strains. *tet*(M) gene as a determinant of resistance to tetracyclines through ribosome protection was detected in 41.6% (57/137) of the examined strains, and the *tet*(S) gene was found in 8% (11/137). The existence of *int-Tn* gene for the integrase of Tn*916*-Tn*1545* was observed in 26.3% (36/137) of strains harboring *tet*(M). Other tetracycline resistance genes (*tet*K and *tet*L) were not found in the *L. monocytogenes* strains.

Most (82.8%, 24/29) strains isolated from farm I harbored *qnrS* gene, whereas most strains from farms II (98.5%, 65/66) and III (100%, 42/42) had *bla*CTX-M gene.

### Genetic diversity of *L. monocytogenes*﻿ strains

The electrophoretic profile of DNA fragments obtained from 137 *L. monocytogenes* strains following ERIC-PCR amplification produced 1–8 bands, whose size ranged from 163 to 3074 bp. The visual comparison of the banding patterns showed 22 distinct ERIC profiles (A–V). The most common ERIC type (Table 5) was ERIC E (10.2%, 14), followed by ERIC F (9.5%, 13), ERIC M (8.8%, 12), ERIC Q (7.3%, 10), ERIC A,C,J,L (6.6%, 9), and ERIC G,I (5.1%, 7), ERIC T (4.4%, 6), ERIC V (3.6%, 5), ERIC D,K,S (2.9%, 4), ERIC P,R (2.2%, 3), ERIC B,N,O,U (1.4%, 2), and ERIC H (0.7%, 1).

**Fig. 1:**
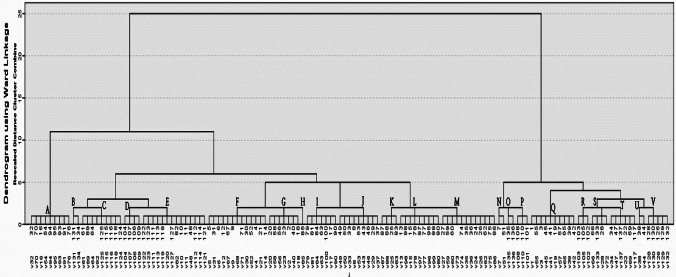
Dendrogram representing genetic relationships between *L. monocytogenes* isolates based on ERIC-PCR fingerprints. Twenty-two ERIC profile represented by A–V and the isolates ID represented by 1–136

The ERIC-PCR dendrogram (Fig. 1) showed that *L. monocytogenes* obtained from the three farms were genetically diverse and heterogeneous, as indicated by their categorization into specific genotype by sampling site and sample type. The ecological distribution of the 22 ERIC types of *L. monocytogenes* in the examined farms is shown in Table 5. Furthermore,13, 16, and 12 ERIC types were observed in farm I (A, E–G, I–N, M, Q, S, and T), farm II (A, C, E–N, Q, and S–U), and farm III (B–E, I, L, O, P, R–T, and V). Only ERIC E, I, L, S, and T were common in the three farms. ERIC A, E, F, G, I–N, Q, and S–T were present in farms I and II. ERIC C, E, I, L, S, and T were found in farms II and III. In farm I, the predominant type was ERIC F (24%, 7/29), followed by ERIC G (13.8%, 4/29) at which ERIC F was common in milk, feces, silage, and BTM isolates, whereas ERIC G was common in the isolates of feces and silage only. The predominant type in farm II was ERIC M (13.6%, 9/66), followed by ERIC A (12.1%, 8/66), ERIC J (10.6%, 7/66), ERIC Q (10.6%, 7/66), ERIC F (9%, 6/66), ERIC I (7.6%, 5/66), and ERIC C (6%, 4/66), which accounted for 69.7% (46/66) of the isolates in farm II (Table 5). ERIC A was common in isolates of milk, feces, and silage; ERIC F and C were common in feces and silage isolates; and ERIC M was common in milk, manure, and BTM isolates. The five predominant types in farm III were ERIC E (23.8%, 10/42), ERIC C (11.9%, 5/42), ERIC V (11.9%, 5/42), ERIC D (9.5%, 4/42), and ERIC L (9.5%, 5/42). ERIC E and C were evident in the isolates of faces and silage. ERIC V was found in the isolates of milk, manure, and floor swabs of the milking room. It was also common in fecal and water isolates.

## Discussion

The epidemiology of *L. monocytogenes* in clinical human, animal, and food specimens has been widely explored (Castro et al. 2018; Swetha et al. 2021). To our knowledge, this study was the first to elucidate the ecology of *L. monocytogenes* in dairy cattle farms and identify the sources of the pathogen and risk factors in the environment of dairy cattle farms. Various samples were collected from clinically normal cattle, the environment, and the milking system. The results showed that the prevalence of *L. monocytogenes* was 7.23% (farm I, 3.4%; farm II, 15.2%; farm III, 6.9%) in all the three studied farms. Mohammed and Abdel Aziz (2017) found a greater prevalence (28.1%) in Egypt possibly because of differences in farm size, management practices, and hygiene ranking. Conversely, the prevalence of *L. monocytogenes* was low in the USA (4.48%) (Van Kessel et al. 2011), Iran (2.02%) (Sohrabi et al. 2013), and Italy (1.6%) (Bianchi et al. 2013).

The prevalence of *L. monocytogenes* was higher in the environment than in dairy cattle. Moreover, silage (27.8%) and manure (19.4%) followed by water and soil (8.3%, each) were considered the principal source of *L. monocytogenes* in the dairy farm environment. These values confirmed the importance of environmental sources of *L. monocytogenes* in dairy farms, especially silage (Mohammed and Abdel Aziz 2017). The prevalence of *L. monocytogenes* in milk filters was higher (6.7%) than that in the BTM samples (5.2%) partly because of the concentrations of the bacteria in the filter and dilution in the tanks (Vilar et al. 2007; Bandelj et al. 2018).

Data analysis showed that five risk factors were significantly (*P* < 0.05) associated with the increased shedding of *L. monocytogenes* in dairy cattle farms. These risk factors included two environmental factors (season and hygienic condition in the farms) and three animal factors (age of cows, parity, and milk yield). The prevalence of *L. monocytogenes* had significant seasonal variation in all types of samples (animal, environmental, and milking equipment samples) from the three examined farms. In particular, the prevalence was higher in winter than in spring and summer. Although the analysis of seasonal variation was limited by the short study period, this finding was consistent with those of other investigations (Dalzini et al. 2016; Bandelj et al. 2018) which identified significant seasonal differences and revealed the higher prevalence of *L. monocytogenes* in cold seasons (winter and early spring) than in other seasons. This high prevalence in cold months could be due to several factors, including the crowding of cattle in indoor facilities, difficulty in maintaining excellent hygiene practices under such circumstances, and the ingestion of spoiled silage (Ryser and Marth, 2007). Other studies have identified insignificant seasonal differences (Hassan et al. 2001; Mohammed et al. 2010). This disparity in seasonal prevalence among different studies might be attributed to variations in study design, climate, and management practices in various geographical regions.

The environmental prevalence of *L. monocytogenes* in farm II was higher than that in farms I and III. As such, it could be due to poor milking hygiene and contamination pressure from the environment of farms to dairy cattle in our study (Castro et al. 2018).

In the three farms studied, the fecal shedding of *L. monocytogenes* had a positive significant relationship with the age, parity, and milk yield of dairy cattle. Dairy cattle are affected by several stressors, such as pregnancy, parturition, and lactation, which may suppress host immunity, increase *L. monocytogenes* fecal shedding in healthy ruminants, and increase their susceptibility to pathogen infection (Roberts and Wiedmann 2003).

The presence of key virulence factors, hlyA and prfA, confers pathogenicity to *L. monocytogenes* strains (Poimenidou et al. 2018). The *hlyA*, which encodes listeriolysin O (LLO), is a basic *L. monocytogenes* pathogenicity gene that helps release bacterial cells through the host cell vacuole (Roberts et al. 2005). PrfA is a protein required for the transcription of the prfA-regulated virulence gene cluster and prfA itself. The iap is a surface protein that acts as a murein hydrolase. In our study, the majority of *L. monocytogenes* strains (93) were positive for *hlyA* and *prfA* although 44 tested strains were positive for only one virulence gene (*hlyA* or *prfA*). Conversely, all the tested bacterial strains were negative for *iap*. These virulence genes are linked to *L. monocytogenes* strains from clinical and food samples in Ireland (Poimenidou et al. 2018), animals in Egypt (Elbar et al. 2020), and the environment in South Africa (Iwu and Okoh 2020). Consistently, the rates of *prfA* and *hly* in *L. monocytogenes* strains obtained from environmental water in South Africa are high (Kayode et al. 2021). Therefore, the presence of the virulence genes strongly suggested that *L. monocytogenes* strains from the studied dairy cattle farms could cause listeriosis in humans.

MAR *L. monocytogenes* from different sources, such as human, food, and environmental samples, has been widely described (Castro et al. 2018; Swetha et al. 202). In this work, all *L. monocytogenes* strains from dairy farms were resistant to penicillin, neomycin, cefoxitin, and nalidixic acid. They were also resistant to amoxicillin, cloxacillin, cefotaxime, amikacin, erythromycin, norfloxacin, tetracyclines, and gentamicin, which are frequently useful in the treatment of human listeriosis. However, they were highly susceptible to chloramphenicol and ciprofloxacin. These findings confirmed previous observations, which demonstrated that *L. monocytogenes* strains have variable resistance to commonly used antibiotics in the medication of clinical and veterinary infections (Su et al. 2016; Swetha et al. 2021). Tahoun et al. (2017) reported high tetracycline, clindamycin, and rifampicin resistance in *L. monocytogenes* isolated from an Egyptian dairy farm. However, a previous investigation showed that *L. monocytogenes* isolated from environmental water has high resistance rates against sulfamethoxazole, oxytetracycline, and amoxicillin but not against ampicillin (Kayode et al. 2021). These differences in the susceptibility patterns of *L. monocytogenes* strains could be dependent on geographical variations and antibiotic use for humans and animals.

All *L. monocytogenes* strains exhibited multiple resistances to four classes of antibiotics, namely, β-lactam (particularly second-generation cephalosporins), aminoglycosides, quinolones, and macrolides, which pose risks to public health because of challenges in the treatment of listeriosis. Furthermore, 25 antimicrobial resistance patterns were observed among the MAR *L. monocytogenes* strains isolated from dairy farms, viewing resistance to antibiotics ranging from 4 to 14. This observation was consistent with the findings of Iwu and Okoh (2020) on higher multiple resistance than single resistance. The observed resistance might be attributed to medication use or feed additives in the livestock industry (Zeitoun et al. 2015). It is significant in the context of the incidence of temporal and spatial changes in antibiotic resistance (Yan et al. 2010). Thus, the emergence of antibiotic resistance should be continuously monitored, and other treatment methods should be developed. In the current work, all *L. monocytogenes* had a MAR index of > 0.20, indicating that the strains isolated from the three dairy farms originated from high-risk sources in which they were constantly exposed to antibiotics and had a high-risk potential (Bilung et al. 2018).

The mechanisms conferring resistance to different classes of antibiotics were explored to determine whether each MAR *L. monocytogenes* strain in this investigation had at least one antimicrobial resistance gene. The results showed more prevalence of *bla*CTX-M gene responsible to produce CTX-M β-lactamases among cefotaxime-resistant strains in comparison to the gene encoding DHA-type β-lactamases (Iwu and Okoh 2020). The first *L. monocytogenes* strains isolated from dairy farms harboring the plasmid-mediated AmpC β-lactamase DHA-1 gene were designed a few years after the statement of gram-negative bacteria harboring the *bla*DHA-1 gene*.* The widespread use of β-lactam antibiotics has resulted in a surge in the occurrence rate of ESBLs because of their low toxicity and effectiveness in the treatment of various infectious diseases, thereby posing a severe threat to global health (Livermore 1996).

Plasmid-mediated quinolone resistance genes (*qnrS*, *qnrA*, and *qnrB*) and *parC* were observed in quinolone-resistant strains. Though quinolones are not recommended treatment options for Listeria infections, they can indirectly disseminate the emergence of resistant *L. monocytogenes* strains because of their massive usage for the medication of multiple infections (Godreuil et al. 2003). The resistance of Gram-positive bacteria to quinolones is due to adjustments in the quinolone resistance–determining regions of the intracellular targets of quinolones, DNA gyrase encoded by *gyrA* and *gyrB*, and topoisomerase IV encoded by *parC* and *parE* (Hooper and Jacoby 2016). The *erm* (erythromycin ribosome methylase) encodes a 23S rRNA methyltransferase responsible for the modification of the macrolide–lincosamide–streptogramin B (MLSB) antibiotic binding site (Leclercq 2002).

Three MLSB resistance genes, namely, *erm*B, *erm*C, and *msr*A, were determined in the erythromycin-resistant strains. Among them, *ermB* is the most predominant (Morvan et al. 2010). However, *erm* (A), *erm* (TR), and *mef* (A) encoding the recorded efflux pumps in Gram-positive bacteria were not observed in the tested strains (Leclercq 2002; Granier et al. 2011). The high occurrence of *dfrD*, encoding a resistant dihydrofolate reductase, has been reported in *L. monocytogenes* strains from the environment and humans (Morvan et al. 2010). In our study, the prevalence of *tet* (M) was higher than that of *tet* (S) that was also noticed by Escolar et al. (2017). The MAR strains exhibited resistance to tetracycline, indicating ribosome protection because of *tet*(M) or *tet*(S) (Charpentier and Courvalin 1999). As a determinant of resistance to tetracyclines, *tet* (M) is highly prevalent in Gram-positive bacteria resistant to tetracyclines and commonly related to conjugative elements of the Tn*916* family (Leclercq et al. 2005). In our study, the existence of *int-Tn* gene in the strains harboring *tet* (M) confirmed that *L. monocytogenes* was partly resistant to tetracycline because of the acquisition of conjugative transposons (Poyart-Salmeron et al., 1989).

ERIC-PCR analysis revealed that the *L. monocytogenes* strains from the three dairy farms were genetically diverse and heterogeneous. Heterogeneity was indicated by the various origins of the strains (animal, environment, and milking equipment) and sampling locations. The potential contamination routes of *L. monocytogenes* in the dairy cattle farm environment were detected by investigating the prevalence of genotypes among the three farms and sampling areas inside each farm through ERIC-PCR. In the three farms, the sources of predominant genotypes were milk, feces, silage, manure, floor swabs of the milking room, and water, suggesting that various locations in the farm environment could be considered ecological niches for the persistence of *L. monocytogenes* inside dairy cattle farms (Morvan et al. 2010). This study suggested that the consumption of contaminated silage was the potential source of *L. monocytogenes* in the investigated farms (Nightingale et al. 2005; Ho et al. 2007). *L. monocytogenes* contamination in BTM was likely attributed to the milking system (teat cups and milking filters), as observed in other studies (Terentjeva et al. 2021). Our study also demonstrated low similarity in the *L. monocytogenes* genotypes among the three farms possibly because of the geographical location of the three farms (Dakahlia Governorate). Moreover, strains with the same genotype had diverse antibiotic susceptibility. For example, the strains (*n* = 7) with a similar ERIC A type had different antibiotic resistance patterns, antibiotic resistance genes, and virulence genes.

## Conclusion

For the first time, this work investigated the prevalence of virulent and multi-antibiotic-resistant *L. monocytogenes* strains in dairy cattle farms in Egypt. Genotyping analysis through ERIC-PCR revealed that all *L. monocytogenes* strains were diverse and heterogenous, as they were categorized into specific genotypes in terms of sampling sites and sample type. Our study confirms the importance of hygienic practices with respect to silage production and milking hygiene should be developed and implemented to prevent not only the introduction and spread of *L. monocytogenes* into herds but also its entry in milk. These findings elucidated the epidemiology of *L. monocytogenes* in dairy cattle farms and served as a basis for implementing control strategies to reduce the risks of *L. monocytogenes* dissemination in dairy cattle and the environment.

## Supplementary Information

Below is the link to the electronic supplementary material.

Supplementary file1 (DOC 267 KB)

## Data Availability

All data generated or analyzed during this study are included in this published article and its supplementary information files.
