# Supplementary material for: Epidemiological study on Listeria monocytogenes in Egyptian dairy cattle farms’ insights into genetic diversity of multi-antibiotic-resistant strains by ERIC-PCR
Source: Environ Sci Pollut Res Int. 2022 Mar 17;29(36):54359–77. doi: 10.1007/s11356-022-19495-2 (PMC9356925; doi:10.1007/s11356-022-19495-2)
Supplement: Supplementary file 1 — Supplementary file1 (DOC 267 KB) [file 11356_2022_19495_MOESM1_ESM.doc]

| **Farm III** | **Farm II** | **Farm I** | 1. **General farm information** |
| --- | --- | --- | --- |
| Dekernes | Aga | Gamasa | Location |
| 140 | 100 | 200 | Herd size |
| Silage and concentrate | Silage and concentrate | Silage and concentrate | Feeding type |
| Outside | Inside | Outside | Vehicle parking for visitors (inside or outside the farm) |
| Other cattle farm away by 1.5 Km | There is cattle farm within 1 Km | Next cattle farm away by 3 Km | Cattle farms within 1 km |
| No | No | No | External rearing |
| No | Yes | No | Sheep/goats in the farm |
| No | Yes | No | Shared materials (ear tag applicators, calving materials, cleaning materials or others) |
| Yes | No | Yes | Veterinary supervision |
|  | | | 1. **Animals’ movements** |
| Germany | Germany and Egypt | Germany | Origin of animals |
| There is isolation room | Isolation done in separate place in the south of the farm | There is isolation room for newly purchased and diseased animals | Quarantine facilities |
| Burial without any disinfectants beside the farm | Throw in water source | Burial using quick lime  Away the farm | Disposal of dead animals |
|  | | | 1. **Purchase of animals (heifers/cows)** |
| Yes | Yes | Yes | Testing on arrival |
| Yes | No | Yes | Quarantine |
|  | | | 1. **Visitors and staff** |
| No | No | Yes | Using of protective clothes |
| No | Yes | No | Visitors may contact animals |
|  | | | 1. **Health status** |
| Penicillin, Amoxycillin, Enrofloxacin, Tylosin, Florfenicol | Penicillin, Amoxycillin,  Gentamycin,  Florfenicol, lincospectin | Penicillin, Amoxycillin, Gentamycin, Tylosin, Enrofloxacin and Sulfa+trimethoprim | Antibiotics |
| Routine vaccination to respiratory and enteric pathogen | | | Vaccination |
|  | | | 1. **Milking process** |
| Mechanical (Herring bone milk parlor) | Mechanical  Tandem parlor | Mechanical (Herring bone milk parlor) | Type of milking system |
| Yes | No | yes | Identiﬁcation of animals with mastitis |
| Yes, begin with non-mastitic cows, sublinically mastitic cows and at the end clinically mastitic cows | No | Yes, begin with non-mastitic cows, sublinically mastitic cows and at the end clinically mastitic cows | Correct Milking order |
| Pre-dipping only | No | Yes | Pre and post – dipping |
| No | Yes | Yes | Check that cows lie down after milking |
| Minor deficits in hygiene | Major deficits in hygiene | No notable deficits in hygiene | Hygiene in milk room, milking station and waiting area |

**Supplementary Table (1). Structured questionnaire for the examined farms.**

**Supplementary Table (2). Target antibiotic resistance genes, Primers sequences, amplicon sizes.**

| **Antimicrobial Class** | **Target gene** | **Primers sequences** | **Reference** |
| --- | --- | --- | --- |
| [**β-lactam**](https://en.wikipedia.org/wiki/Beta-lactam_antibiotic)**ases** | *blaCTX-M* | F: SCSATGTGCAGYACCAGTAA  R: ACCAGAAYVAGCGGBGC | Ojdana et al. (2014) |
|  | *blaDHA-1* | F: CCAGAATCACAATCGCCACC  R: TATCAGCAGTGGCAGCCGT | Guo et al. (2012) |
|  | *bla*SFO-1 | F: ATTCAGCAGCAACTGTCCG  R: ACGCTTATCGCTGGGAAT | Muratani et al. (2006) |
| **Quinolones** | *qnrA* | F: GGGTATGGATATTATTGATAAA  R: CTAATCCGGCAGCACTATTA | López et al. (2011) |
|  | *qnrB* | F: GGMATHGAAATTCGCCACTG  R: TTTGCYGYYCGCCAGTCGAA |  |
|  | *qnrS* | F: AGTGATCTCACCTTCACCGC  R: CAGGCTGCAATTTTGATACC |  |
|  | *gyrA* | F: AGTGTAATTGTTGCCCG  R: ATATCGCCATCAACCGA | Godreuil et al. (2003) |
|  | *ParC* | F: GAACGTGCGCTTCCAGA  R: GTTGCATAACCAGCGGA |  |
| **Macrolides** | *erm(A)* | F: CTTCGATAGTTTATTAATATTAGT  R: TCTAAAAAGCATGTAAAAGAA | **Morvan et al. (2010)** |
|  | *erm(B)* | F: GAAAAGGTACTCAACCAAATA  R: AGTAACGGTACTTAAATTGTTTAC |  |
|  | *erm(C)* | F: TCAAAACATAATATAGATAAA 641  R: GCTAATATTGTTTAAATCGTCAAT |  |
|  | *erm(TR)* | F: GAAGTTTAGCTTTCCTAA  R: TTTCCACCATTAACA |  |
|  | *msr(A)* | F: GCAAATGGTGTAGGTAAGACAACT  R: ATCATGTGATGTAAACAAAAT |  |
|  | *mef(A)* | F: AGTATCATTAATCACTAGTGC 345  R: TTCTTCTGGTACTAAAAGTGG |  |
| **Trimethoprim** | *dfrD* | F: AGAGTAATCGGCAAGGATAACG  R: AATGGGCAATTTCACAATCC |  |
| **Tetracyclines** | *tet(K)* | F: CGATAGGAACAGCAGTATGG  R: TTAGCCCACCAGAAAACAAACC |  |
|  | *tet(L)* | F: CCACCTGCGAGTACAAACTGG  R: TCGGCAGTACTTAGCTGGTGA |  |
|  | *tet(M)* | F: GTGGACAAAGGTACAACGAG  R: CGGTAAAGTTCGTCACACAC |  |
|  | *tet(S)* | F: ATCAAGATATTAAGGAC  R: TTCTCTATGTGGTAATC |  |
|  | *Int-Tn* | F: GATGGTATTGATGTTGTAGG  R: GGTCTATATATTGACAAGACCG |  |

**Supplementary Table (3): Antibiotic resistance pattern of multi-resistant *L. monocytogenes* strains in the dairy cattle farms**

| **Antibiotics pattern profile** | **Antibiotics** | **No. of resistance antibiotics** | **No. of isolates (%)** | **MARI (%)** |
| --- | --- | --- | --- | --- |
| **1** | **P, N, FOX, NA** | **4** | **4** | **0.22** |
| **2** | **P, N, FOX, NA, CTX** | **5** | **4** | **0.28** |
| **3** | **P, N, FOX, NA, AML** | **5** | **4** | **0.28** |
| **4** | **P, N, FOX, NA, AML, OB, CN** | **7** | **5** | **0.39** |
| **5** | **P, N, FOX, NA, AML, OB, CTX, CN** | **8** | **2** | **0.44** |
| **6** | **P, N, FOX, NA, OB, AK, E, SXT, CIP** | **9** | **10** | **0.5** |
| **7** | **P, N, FOX, NA, OB, CTX, AK, E, CIP** | **9** | **4** | **0.5** |
| **8** | **P, N, FOX, NA, AML, OB, CTX, AK, E, CN** | **10** | **2** | **0.56** |
| **9** | **P, N, FOX, NA, AML, OB, CTX, AK, E, S** | **10** | **7** | **0.56** |
| **10** | **P, N, FOX, NA, AML, OB, CTX, NOR, SXT** | **9** | **5** | **0.5** |
| **11** | **P, N, FOX, NA, OB, CTX, AK, E, S, CIP** | **10** | **6** | **0.56** |
| **12** | **P, N, FOX, NA, OB, CTX, NOR, AK, E, CN** | **10** | **5** | **0.56** |
| **13** | **P, N, FOX, NA, OB, CTX, NOR, AK, E, TE** | **10** | **3** | **0.56** |
| **14** | **P, N, FOX, NA, AML, OB, CTX, AK, E, CN, S** | **11** | **2** | **0.61** |
| **15** | **P, N, FOX, NA, AML, OB, CTX, NOR, AK, E, S** | **11** | **4** | **0.61** |
| **16** | **P, N, FOX, NA, OB, CTX, NOR, AK, E, TE, CN** | **11** | **3** | **0.61** |
| **17** | **P, N, FOX, NA, OB, CTX, NOR, AK, E, TE, S** | **11** | **2** | **0.61** |
| **18** | **P, N, FOX, NA, AML, OB, CTX, NOR, AK, E, TE, S** | **12** | **1** | **0.67** |
| **19** | **P, N, FOX, NA, OB, CTX, NOR, AK, E, TE, CN, S** | **12** | **2** | **0.67** |
| **20** | **P, N, FOX, NA, OB, CTX, NOR, AK, E, TE, CN, SXT** | **12** | **6** | **0.67** |
| **21** | **P, N, FOX, NA, AML, OB, CTX, NOR, AK, E, TE, S, CIP** | **13** | **10** | **0.72** |
| **22** | **P, N, FOX, NA, OB, CTX, NOR, AK, E, TE, CN, SXT, C** | **13** | **9** | **0.72** |
| **23** | **P, N, FOX, NA, OB, CTX, NOR, AK, E, TE, CN, S, CIP** | **13** | **4** | **0.72** |
| **24** | **P, N, FOX, NA, OB, CTX, NOR, AK, E, TE, CN, SXT, S, CIP** | **14** | **23** | **0.78** |
| **25** | **P, N, FOX, NA, OB, CTX, NOR, AK, E, TE, CN, SXT, S** | **14** | **10** | **0.78** |

**
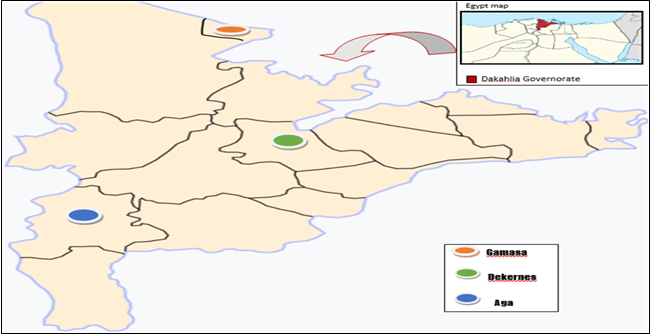
**

**Supplementary figure (1).** Map of Dakahlia governorate showing the location three selected dairy cattle farms for the study in relation to the rest of Dakahlia (orang circle represents farm I located in Gamasa city, green circle represents farm II located in Dekernes city while blue circle points out to Farm III located in Aga).
